# Strain specific maturation of Dendritic cells and production of IL-1β controls CD40-driven colitis

**DOI:** 10.1371/journal.pone.0210998

**Published:** 2019-01-17

**Authors:** Ana Ogrinc Wagner, Verena Friedrich, Christian Barthels, Peggy Marconi, Andreas Blutke, Frank Brombacher, Thomas Brocker

**Affiliations:** 1 Institute for Immunology, Faculty of Medicine, Ludwig-Maximilians-Universität München, Planegg-Martinsried, Germany; 2 Department of Chemical and Pharmaceutical Sciences, University of Ferrara, Ferrara, Italy; 3 Research Unit Analytical Pathology, Helmholtz Zentrum München, Neuherberg, Germany; 4 Division of Immunology, University of Cape Town & South African Medical Research Council, Cape Town, South Africa; 5 International Centre for Genetic Engineering and Biotechnology, Cape Town component, South Africa; Mie Daigaku, JAPAN

## Abstract

Intestinal integrity is maintained by balanced numbers of CD103^+^ Dendritic cells (DCs), which generate peripherally induced regulatory T cells (iTregs). We have developed a mouse model where DC-specific constitutive CD40 signals caused a strong reduction of CD103^+^ DCs in the lamina propria (LP) and intestinal lymph nodes (LN). As a consequence, also iTregs were strongly reduced and transgenic mice on the C57Bl/6-background (B6) developed fatal colitis. Here we describe that transgenic mice on a pure Balb/c-background (B/c) do not show any pathologies, while transgenic C57Bl/6 x Balb/c (F1) mice develop weak colon inflammation, without fatal colitis. This graded pathology correlated with the effects of CD40-signalling on DCs in each background, with striking loss of CD103^+^ DCs in B6, but reduced in F1 and diminished in B/c background. We further show direct correlation of CD103^+^ DC-numbers with numbers of iTregs, the frequencies of which behave correspondingly. Striking effects on B6-DCs reflected robust loss of surface MHCII, known to be crucial for iTreg induction. Furthermore, elevated levels of IL-23 together with IL-1, found only in B6 mice, support generation of intestinal IFN-γ^+^IL-17^+^ Th17 cells and IFN-γ^+^ Th1 cells, responsible for onset of disease. Together, this demonstrates a novel aspect of colitis-control, depending on genetic background. Moreover, strain-specific environmental sensing might alter the CD103^+^ DC/iTreg-axis to tip intestinal homeostatic balance to pathology.

## Introduction

FoxP3^+^ regulatory T cells (Tregs) control immune responses and maintain homeostatic immunological balance in many tissues and organs [1]. Tregs can develop in the thymus as natural regulatory T cells (nTregs) or in peripheral tissues by differentiation from mature CD4^+^ T cells to induced Tregs (iTregs) [2]. This latter process requires transforming growth factor β (TGFβ) as well as the presence of microbiota [3]. iTregs occur mainly in intestinal tissues, where Dendritic cells (DCs) present dietary and commensal antigens and play a central role in mucosal homeostasis [4]. Microbiota induce expression of retinoic acid receptor-related orphan γt (RORγt) in iTregs [5, 6]. The deletion of iTregs caused increased production of inflammatory cytokines and raised the susceptibility of mice to develop inflammatory bowel disease (IBD) [5, 6].

Especially CD103^+^ DCs take up gut luminal antigens and migrate from the lamina propria (LP) to the regional lymph nodes (LN) for induction of iTregs [7–9]. CD103^+^CD11b^+^ DCs have major tolerogenic properties but can also induce Th17 and Th1 cells upon proper stimulation [10–13]. Also, CD103^+^CD11b^-^ DCs express high levels of proteins necessary for induction of iTregs, such as aldehyde dehydrogenase (ALDH) and TGFβ [14]. LP-resident CD103^-^CD11b^+^ DCs rather resemble macrophages but can induce Th17 and Th1 cells without further stimulation [14].

We have recently published a novel mouse model, where the DC–iTreg axis was manipulated and mice spontaneously developed colitis due constitutive CD40 signaling in CD11c^+^ DCs [15]. Intestinal CD103^+^ DCs expressed a constitutively active LMP1/CD40-transgene, upregulated CCR7 and migrated from LP to LNs where they died by apoptosis causing attrition of CD103^+^ DCs in LP and mesenteric (m)LNs [15]. This resulted in deficiency to induce RORγt^+^ iTregs and increased IL-17^+^IFNγ^+^ Th17/Th1 cells and pathogenic IFNγ^+^ Th1 cells [15].

Importantly, this model is of direct relevance to the human IBD situation, where the CD40-CD40L axis is also key to disease. T cells and platelets of IBD patients express higher levels of CD40L and their serum levels of soluble CD40L are elevated [16–18]. Indeed, a single nucleotide polymorphism in the human CD40 promoter region was identified to play a mechanistic role in disease susceptibility of IBD [19]. Moreover, in patients with Crohn’s disease CD40 is overexpressed on mucosal cells, endothelial cells and DCs [20, 21]. Accordingly, administration of blocking CD40L antibodies could inhibit cellular infiltration and colitis in mice [22, 23]. Furthermore, the treatment of Crohn’s disease patients with antagonistic CD40 antibody showed beneficial responses and high remission rates [24]. Taken together, CD40-signalling is involved in colitis and IBD and we show in the present study that the role for CD40-signaling in colitis depends on strain variations. While loss of CD103^+^ DCs and disappearance of iTregs are strongest in mice of the C57BL/6(B6)-background, they are very modest in the Balb/c(B/c)-background and intermediate in F1 (B6 x B/c) mice. CD40-induced lack of iTregs correlates strongly with reduction of CD103^+^ DCs themselves as well as their expression of MHCII, which is also graded in different backgrounds. Furthermore, the production of IL-1 in B6, but not F1 and B/c mice of this model contributes to strain-specific differential effector CD4^+^ T cell accumulation in the colon. Our data indicate that CD40-signalling in colitis is not a single on/off switch, but that additional determinants contribute to the control of the DC-iTreg axis in CD40-mediated colitis.

## Materials and methods

### Mice

To obtain F1DC-LMP1/CD40 animals, B6CD11c-Cre mice (Tg(Itgax-cre)1-1Reiz [25]) were crossed with B/cLMP1/CD40^flstop^ mice (B/c-Gt(ROSA)26Sortm1Uzs [26]). B/cLMP1/CD40flstop mice were backcrossed to the B6-background for more than 10 generations to obtain B6LMP1/CD40flstop mice. Those were crossed with B6CD11c-Cre to obtain B6DC-LMP1/CD40 mice [15]. B/cCD11c-Cre mice [27] were bred to B/cLMP1/CD40flstop mice to obtain B/cDC-LMP1/CD40 animals. Mice were analyzed in age-matched groups of 8–12 weeks of age. Littermate animals were used as controls in a non-randomized, non-blinded fashion and mice were sacrificed by cervical dislocation. The SPF-status of the facility was tested according to the Federation for Laboratory Animal Science Associations (FELASA) recommendations. Animal experiment permissions were granted by animal ethics committees Regierung von Oberbayern, Munich, Germany and Organismo preposto al benessere animale di Universita di Ferrara, Italy. All mice were bred and maintained at the animal facility of the Institute for Immunology, Ludwig-Maximillians-Universität München. Antibody treatment was performed at the Department of Life Sciences and Biotechnology, University of Ferrara.

### Single-cell preparation

Single-cell suspensions of lymph nodes were prepared by meshing organs through a 100 μM cell strainer. Samples were washed with PBS and stored on ice for further analysis. Number of living cells was determined using CASY Counter (OMNI Life Science). To analyze cells from the lamina propria, the colon was removed, cleaned, opened longitudinally and cut into 5 mm pieces for incubation in (HBSS)-EDTA for 10 min at 37°C. Pieces were digested 3 times for 30 min with a mixture of Collagenase IV (157 Wuensch units/ml, Worthington), DNAse I (0.2 mg/ml, Roche) and Liberase (0.65 Wuensch units/ml, Roche). The supernatant was collected after each step of digestion and cells were washed once with PBS. Collected cells were enriched for immune cells with a 40–80% Percoll gradient centrifugation for 20 min, 1800 rpm, 4°C without break. Cells were collected, washed, counted and stored on ice for further analysis.

### Flow cytometry analysis

For flow cytometric analysis, 2 × 10^6^ cells per staining were plated into a 96 well plate. Cells were stained for 20 min at 4°C in the dark in 50 μl of antibody mix made with antibodies in PBS, 2% FCS, 0.01% NaN3 (FACS buffer). Dead cells were excluded using Aqua LIVE/DEAD Fixable Aqua DeadCell Stain Kit (Invitrogen, TermoFischer, Cat: L34957) or Zombie Aqua Fixable Viability Kit (BioLegend, Cat: 423102). For intracellular stainings, cells were fixed and permeabilized after extracellular staining with a BD Cytofix/Cytoperm (Fixation and Permeabilization Solution, BD Biosciences, Cat: 51-2090KZ). After washing, cells were stained for intracellular markers for additional 30 min. For intra-nuclear staining of FoxP3, Helios and Tbet, cells were washed once and then resuspended in 200 μl 1x Fixation/Permeabilization solution BD Cytofix/Cytoperm (Fixation and Permeabilization Solution, BD Biosciences, Cat: 51-2090KZ) for 20 min at 4°C in the dark. Cells were washed twice with 1x Permeabilization Buffer and stained with a specific antibody in 50 μl 1x Permeabilization Buffer for 30 min at 4°C in the dark. Afterwards, cells were washed once and acquired on BD FACSCanto. Cell sorts were performed on a FACSAriaIII and a FACSAria Fusion (BD, Heidelberg, Germany). The following antibodies were used: CD3 (145-2C11; PE-Cy7, dil. 1:400), CD11b (M1/70; APC-eFluor780, dil. 1:400), CD11c (N418; PE-Cy7, dil. 1:600; APC, dil. 1:100), CD25 (PC61.5; PerCP-Cy5.5, dil. 1:400), CD80 (16-10A1; PE, dil. 1:400), FoxP3 (FJK-16s; eFlour660, dil. 1:50), Helios (22F6; FITC, dil. 1:400), MHCII (M5/114.15.2; FITC, dil. 1:800, PerCP-Cy5.5, dil. 1:800), IFN-γ (XMG1.2; FITC, dil. 1:500; APC, dil. 1:400), Tbet (eBio4B10; PE-Cy7, dil. 1:100), IL-17-A (TC11-18H10.1; PE, dil. 1:200) (eBioscience); CD86 (GL-1; PE, dil. 1:1,000), CD103 (M290; BV421, dil. 1:150; PE, dil. 1:150) (BD Pharmingen); CD4 (GK1.5; APC-Cy7, dil 1:800) (Invitrogen); CD3 (17A2; FITC, dil. 1:400), CD4 (RM4-5; PerCP, dil. 1:800), CD45 (30.F11; APC-eFlour780, dil. 1:200; PerCP, dil. 1:500), CD64 (X54-517.1; APC, dil. 1:200) (BioLegend). Data analysis was performed using FlowJo version 8 and 9 (TreeStar, Ashland, OR, USA).

### In vitro T cell restimulation

To measure cytokine production of T cells 2×10^6^ cells were plated into 96-well plates and stimulated for 4h at 23°C with 40 ng/ml PMA and 1 μg/ml ionomycin in the presence of 2 μM GlogiStop (BD Biosciences, Cat: 554724). Then, cells were washed twice with a FACS buffer and stained for extracellular markers, fixed and stained for intracellular markers.

### Tissue homogenization

For preparation of whole gut protein homogenate samples, 50 mg snap-frozen intestinal tissue samples were homogenized in 500 μl PBS containing a cocktail of protease inhibitors (cOmplete ULTRA Tablets, Roche, Sigma-Aldrich, Cat: 05892953001). For homogenization, a tissue homogenizer (FastPrep-24, MP Biomedicals, Santa Ana, CA, USA) was used (FastPrep speed: 6.0, FastPrep time: 40 sec). Homogenized samples were centrifuged at 9000 rpm for 5 min in order to pellet debris, supernatant was stored at -20°C.

### Cytokine bead array

Whole gut protein homogenate samples were measured with BD Cytokine Bead Array mouse inflammation kit (Cat: No. 552364) according to instructions provided by manufacturer. Samples were diluted 1:1 with an Assay diluent and later acquired on a FACSCantoII. Results were analyzed using FCAP Array Software (Soft Flow Inc.).

### ELISA for IL-1 alpha and IL-1 beta Pro-form

Whole gut protein homogenate samples were diluted 1:10 with an ELISA ELISPOT diluent and measured with an IL-1 alpha ELISA Ready-SET-Go! kit (Affymetrix eBioscience, Cat: 15550857) or with an IL-1 beta Pro-form ELISA Ready-SET-Go! kit (Affymetrix eBioscience, Cat: 15501197) according to manufacturer`s instructions.

### ELISA for fecal lipocalin-2

Fecal samples were reconstituted in PBS containing 0.1% Tween 20 (100  mg/ml) and vortexed for 20  min for homogenisation. Upon centrifugation for 10 min at 12,000 rpm supernatants were analyzed for lipocalin-2 content using Quantikine ELISA kit for mouse Lipocalin-2/NGAL (R&D Systems, Cat: MLCN20).

### Anti IL-1β treatment of B6DC-LMP1/CD40 animals

Treatment with 0.25 mg of anti-IL-1β antibody (clone B122, isotype Armenian hamster IgG, Bio X Cell) or isotype control antibody was performed twice per week i.p. for 7 weeks. Afterwards animals were sacrificed and analyzed.

### Transcriptional analysis

Total RNA from sorted cells was isolated using TRIZOL and cDNA was generated using QuantiTect Reverse Transcription Kit (QIAGEN, Cat No: 205311). TaqMan PCR was performed using the Universal Probe Library Set mouse (Roche) according to manufacturer’s instructions. Gene expression was normalized to HPRT expression. The following Primers were used: HPRT forward 5′-TCCTCCTCAGACCGCTTTT-3′, reverse 5′-CCTGGTTCATCATCGCTAATC-3′, probe # 95; IL-23p19 forward 5′-ATAGCCCCATGGAGCAACTT-3′, reverse 5′- GCTGCCACTGCTGACTAGAA-3′, probe # 25; IL-1a forward 5’-TTGGTTAAATGAC CTGCAACA-3’, reverse 5’-GAGCGCTCACGAACAGTTG-3’, probe # 52; IL-1b forward 5′-TGTAATGAAAGACGGCACACC-3′, reverse 5′-TCTTCTTTGGGTATTGCTTGG-3′, probe # 78. Relative expression was calculated using the ΔΔCt method.

#### Histopathology

Histopathological examination was performed on tissue samples of sex- and age-matched mice. Tissue samples were fixed in 4% neutral buffered formaldehyde solution at room temperature for 24 h and embedded in paraffin. Sections of 3.0 μm thickness were stained with haematoxylin and eosin (HE). All sections were evaluated in a blinded fashion.

#### Statistics

For absolute cell numbers the percentage of living cells of a certain subset was multiplied by the number of living cells as determined by CASY Counter. If not mentioned otherwise, significance was determined using the student´s *t*-test and defined as follows: *P<0.05, **P<0.01, ***P<0.001 and ****P<0.0001. Bar graphs show mean± standard error of mean (SEM). for the group sizes as indicated in the figure legends.

## Results

### Gradual loss of inflammation in DC-LMP1/CD40 mice in B6, F1 and B/c genetic backgrounds

**Fig 1:**
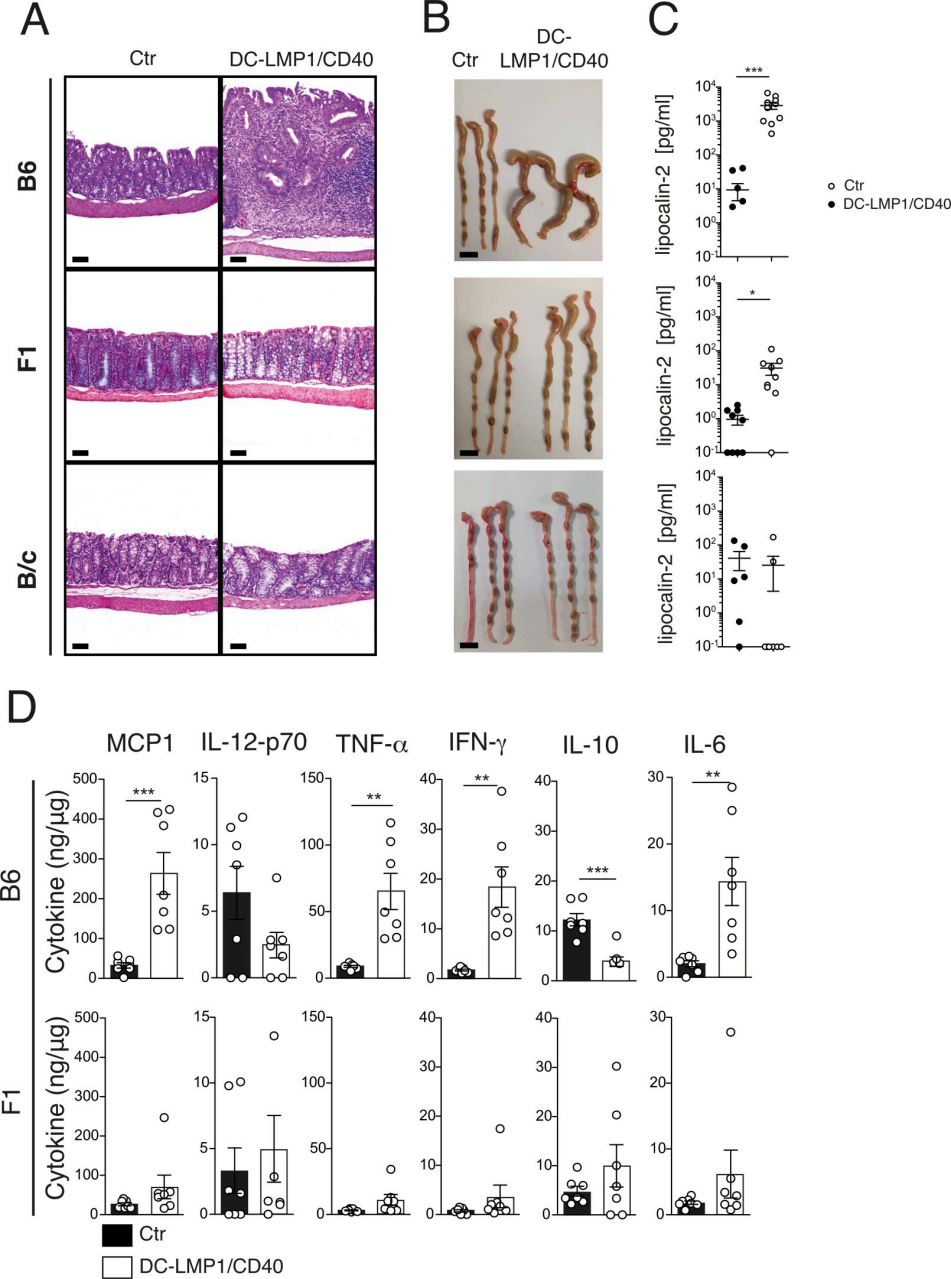
Graded colitis development in DC-LMP1/CD40 mice. (A) B6DC-LMP1/CD40 mice show severe colitis with a thickening of the colon mucosa, extensive proprial infiltration of mixed inflammatory mononuclear cells, loss of crypts and reduction of goblet cells. F1 and B/cDC-LMP1/CD40 mice show no changes in comparison to controls (Ctr). Paraffin sections, HE-staining. Bars = 100μm. (B) Colon pictures from Ctr and DC-LMP1/CD40 animals on different genetic backgrounds. Bars = 1 cm. (C) Levels of fecal lipocalin-2 as measured by ELISA in 8-10-week-old DC-LMP1/CD40 animals on different genetic backgrounds. Shown is pooled data from 3 experiments (n = 6–10). (D) Levels of pro-inflammatory cytokines were measured in colon homogenates from 8-12-week-old B6 and F1DC-LMP1/CD40 mice. Cytokine concentrations were measured with a Cytometric Bead Array (CBA) kit and normalized to the total protein content for each sample. Shown are data from 2 pooled experiments with similar outcome (n = 7). All bar graphs represent mean ± SEM and statistical significance was analyzed using a student´s *t*-test, with *: *P* < 0.05, **: *P* < 0.01 and ***: *P* < 0.001.

To generate the previously published DC-LMP1/CD40 mouse model, we initially bred the original B/c-Gt(ROSA)26Sor^tm1Uzs^ strain to Tg(Itgax-cre)1-1Reiz mice on a B6-background to obtain F1 mice (for short: F1DC-LMP1/CD40). This strain did not show any obvious colitis phenotype (Fig 1A). After nine further backcrosses of the B/cGt(ROSA)26Sor^tm1Uzs^ to the B6-background (for short: B6DC-LMP1/CD40), we observed fatal colitis in all mice as published previously [15](Fig 1A and 1B). Histopathologic examination presented the described phenotype in the LP of B6DC-LMP1/CD40 animals with marked thickening of colon mucosa, extensive proprial infiltration by mixed inflammatory mononuclear cells, loss of crypts, focal cryptitis, ulceration and reduction of goblet cells (Fig 1A). To further study the potential influences of the genetic backgrounds we generated the same strain on a pure B/c-background (B/c-Gt(ROSA)26Sor^tm1Uzs^ x B/c-Tg(Itgax-cre)1-1Reiz) to obtain B/cDC-LMP1/CD40 mice. Similar to F1DC-LMP1/CD40 mice also B/cDC-LMP1/CD40 animals showed no histopathologic alterations (Fig 1A). Colitis was visible macroscopically due to colon shortening and thickening only in mice of the B6-background, but neither in F1- nor B/c-backgrounds (Fig 1B). Measurement of fecal lipocalin-2, a sensitive non-invasive inflammatory marker [28], indicated strong inflammation in B6DC-LMP1/CD40-mice as published previously [15], as well as weak, but significant inflammation in the F1DC-LMP1/CD40 animals (Fig 1C). However, no elevation of lipocalin-2 was observed in mice on the B/c-background (Fig 1C). To further investigate the inflammatory state of colon tissue from F1-mice, we next measured pro-inflammatory cytokines from tissue homogenates (Fig 1D). MCP1, TNF-α, IFN-γ and IL-6 were significantly increased in the colon of mice from the B6-background, while anti-inflammatory IL-10 was significantly reduced (Fig 1D, upper panel). However, although lipocalin-2 was slightly increased in mice from the F1-background (Fig 1C), no change in any cytokines tested could be observed (Fig 1D, lower panel). Therefore, only B6DC-LMP1/CD40-mice showed all signs of inflammation and colitis as published previously [15], while neither F1 nor B/c mice developed colitis, nor did they show signs of inflammatory cytokine production. In general, pathology was strongest in transgenic mice in B6 background, partially present in those of F1-background and absent in B/c mice.

### DC-subset reduction by LMP1/CD40 transgene is background-dependent

**Fig 2:**
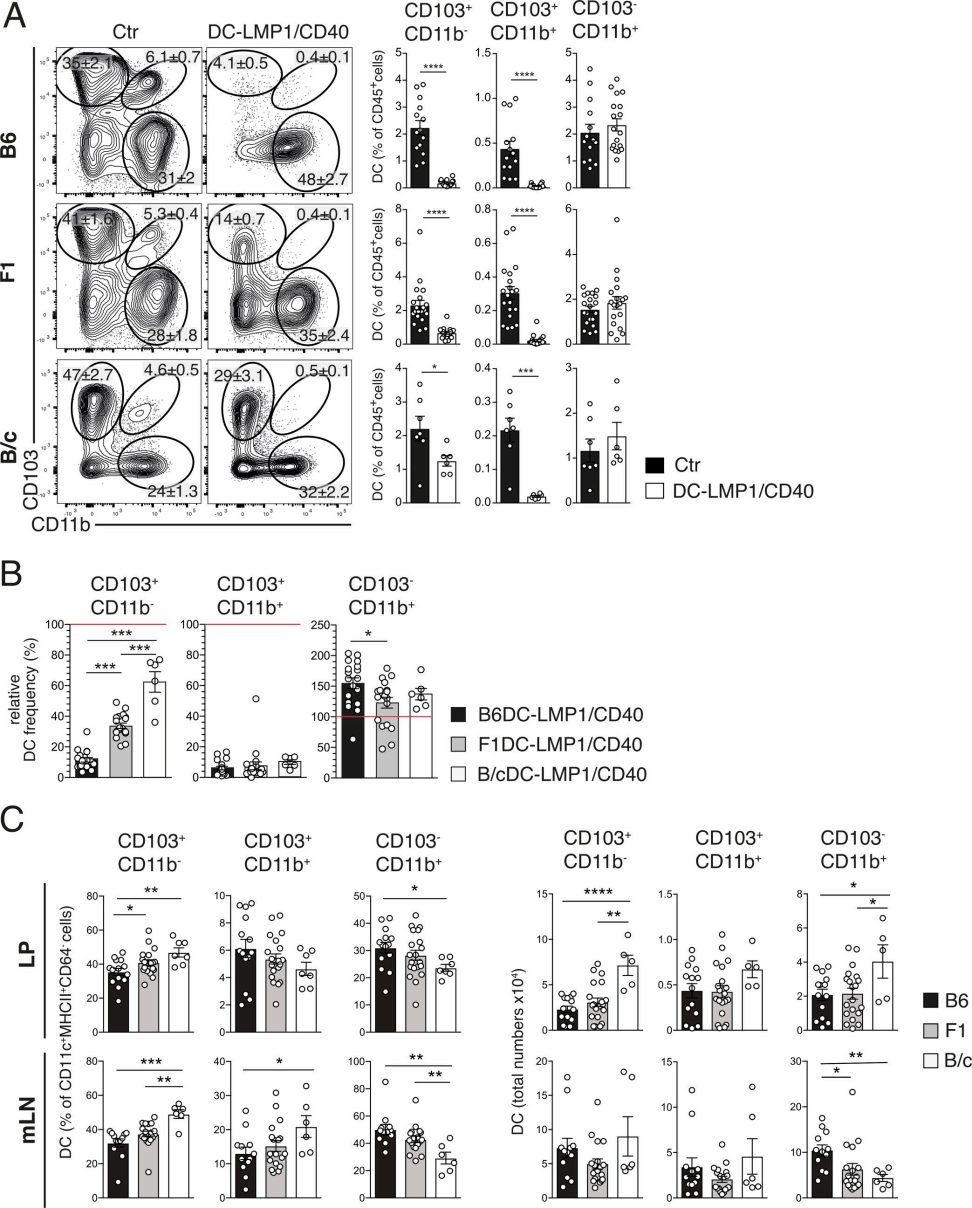
Graded loss of CD103^+^ DCs from the LP of DC-LMP1/CD40 animals. DC subsets in the LP were analyzed in DC-LMP1/CD40 animals on different genetic backgrounds. (A) LP cells were gated on single cells, live, CD45^+^, MHCII^+^CD11c^+^, CD64^-^ cells (see S1A Fig for gating) from control (Ctr) or DC-LMP1/CD40 mice on B6-, F1- or B/c-background. Representative FACS-plots are shown. Numbers in FACS-plots indicate the frequencies of DC subsets within the respective gates. Bar graph shows frequencies of DCs as percent of all CD45^+^ cells. Shown is pooled data from 5 (B6, n = 14–18), 6 (F1; n = 19–20) or 2 experiments (B/c, n = 6–7) with similar outcome. (B) The frequencies for each DC subset in DC-LMP1/CD40 animals (from Fig 2A) on different genetic backgrounds are shown as data relative to the corresponding background control, which was set to 100% (red line). (C) DC subsets in the LP (upper panel) and mLNs (lower panel) were analyzed in wt animals on different genetic backgrounds. Analyses were performed as in (A). Results are displayed as relative DC-frequencies with respect to all DCs (left hand panel) or total DC-numbers (right hand panel). Shown are pooled statistics from 2 experiments with similar outcome (n = 5–6). All bar graphs represent mean ± SEM, significance was analyzed using a student *t*-test, with *: *P* < 0.05, **: *P* < 0.01 and ***: *P* < 0.001.

Tolerogenic CD103^+^ DC subsets are strongly reduced in the intestine of B6DC-LMP1/CD40 animals [15]. To study if the genetic background would influence DC-subsets, we next analyzed DCs from animals of the F1- and B/c-background. The gating strategies for flow cytometry analyses are indicated in S1A–S1C Fig. In colonic lamina propria (LP) the frequencies of CD103^+^CD11b^-^ and CD103^+^CD11b^+^ tolerogenic DCs were reduced in all three DC-LMP1/CD40 strains (Fig 2A). While CD103^+^CD11b^+^ DCs were nearly completely eliminated in DC-LMP1/CD40 mice of all three backgrounds, CD103^+^CD11b^-^ DCs seemed to be differentially affected (Fig 2A). Analysis of DC-subsets in mLNs showed similar effects as in LP (S2 Fig). To better compare DC subsets between different genetic backgrounds, we calculated the reduction of DCs relative to the respective background wildtype (wt) controls, which were set as 100% for each DC subset (Fig 2B). This comparison revealed that the CD103^+^CD11b^-^ DC subset showed approximately 90% reduction of the normal frequency in B6DC-LMP1/CD40 mice, around 60% reduction in F1DC-LMP1/CD40 animals and approximately 40% reduction in B/cDC-LMP1/CD40 mice. Therefore, the overall reduction of CD103^+^CD11b^-^ DCs induced by the LMP1/CD40-transgene was stronger in B6 than F1 and B/c backgrounds. In contrast, CD103^+^CD11b^+^ DC were similarly reduced in all transgenic animals, while CD103^-^CD11b^+^ were increased (Fig 2B). Such graded reduction may be also related to the size of the respective starting populations of CD103^+^CD11b^-^ DCs, which was different. Here B6 mice had the lowest frequencies, F1 mice had slightly higher and B/c had significantly more CD103^+^CD11b^-^ DCs in LP of wt control littermates (Fig 2C). However, these differences were not reflected in the mLNs and could not be found in the other DC-subpopulations, where all mice had comparable DC-frequencies (Fig 2C). Therefore, strain specific factors and DC-frequencies might modulate the effects of LMP1/CD40-signalling causing differential degrees of DC-attrition.

### iTreg reduction by LMP1/CD40 transgene is background-dependent

**Fig 3:**
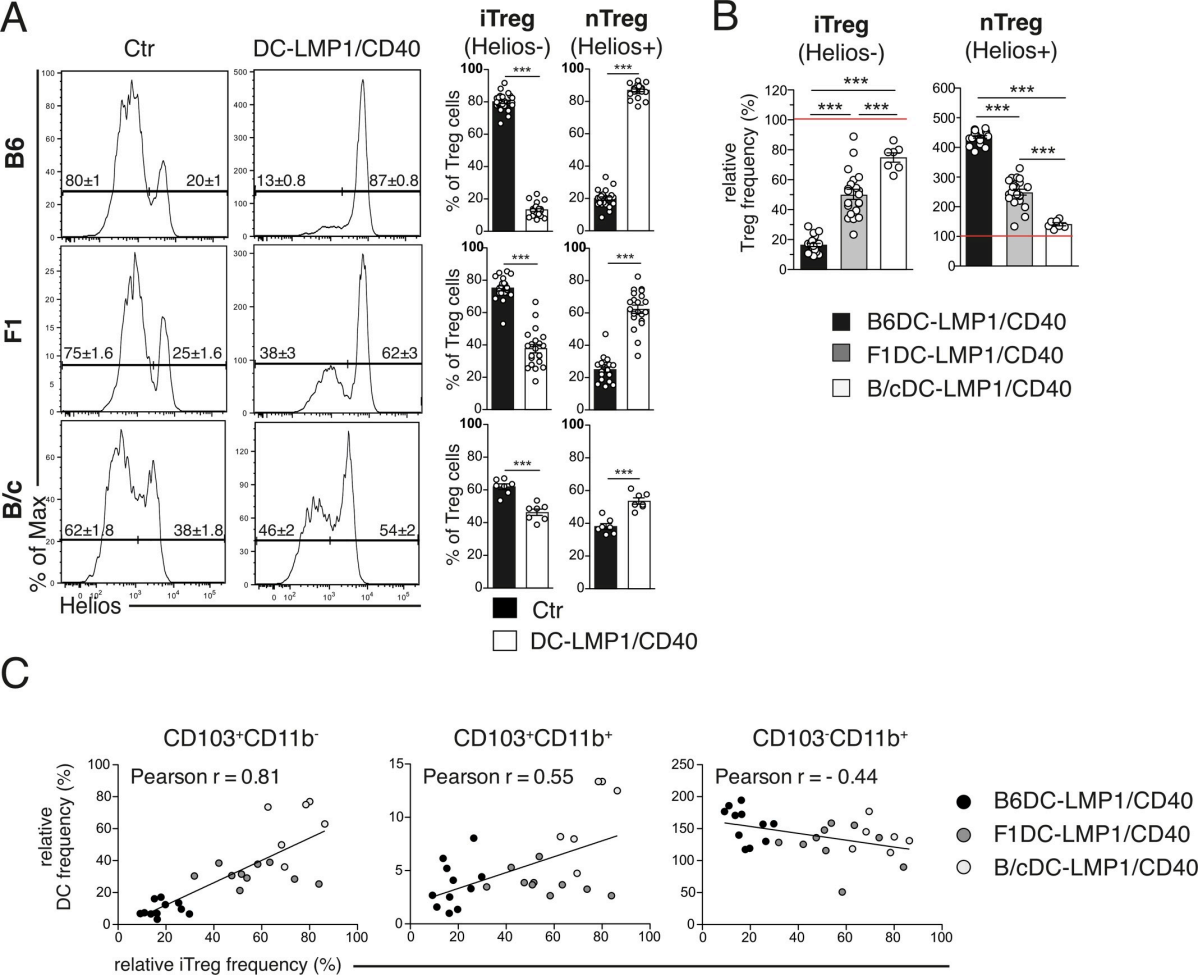
Graded impairment of iTreg induction is background dependent. Single cell suspensions of LP were analyzed for Treg cells. (A) Shown are representative FACS-plots of FoxP3^+^ Tregs found in the LP pre-gated on single, live, CD45^+^CD3^+^CD4^+^CD25^+^FoxP3^+^Helios^+^ nTregs or Helios^-^ iTregs (S1B Fig). Numbers on FACS-plots and bar graphs indicate the frequency of Treg cell subsets, for each genetic background analyzed. Shown are pooled statistics from 7 (B6, n = 23–26), 6 (F1, n = 21) and 2 (B/c, n = 7) experiments with similar outcome. (B) The iTreg and nTreg frequencies from (A) are shown as relative data with the corresponding background control set as 100% (indicated as red line). (C) Relative DC subset (Fig 2B) and iTreg (Fig 3B) frequencies from each mouse were plotted against each other. Each symbol on the scatter plot represents one mouse. A line for linear fit was added on each scatter plot to visualize the relationship between DCs and iTregs. The strength of co-occurrence between DC subpopulations and iTregs is represented by Pearson Correlation coefficient (r), whose values range between -1 and +1. Data for LP were acquired and pooled from 3 (B6, n = 10–12), 4 (F1, n = 13–14) and 2 (B/c, n = 6–7) experiments with similar outcome. All bar graphs represent mean ± SEM and significance were analyzed using a student´s *t*-test, with *: *P* < 0.05, **: *P* < 0.01 and ***: *P* < 0.001.

It has been published previously that CD103^+^ DCs are crucial for iTreg induction [7, 9] and we reported that B6DC-LMP1/CD40 mice had strongly reduced iTreg frequencies in their intestinal tissues due to loss of CD103^+^ DCs [15]. We next analyzed Tregs in the different genetic backgrounds to investigate if their frequencies are directly related to varying numbers of CD103^+^ DCs. When peripheral iTregs (CD4^+^FoxP3^+^Helios^−^) and thymus-generated natural nTreg cells (CD4^+^FoxP3^+^Helios^+^) were analyzed in the LP, only around 13% of all Tregs were iTregs in B6DC-LMP1/CD40 mice in comparison to 80% in non-transgenic B6-controls (Fig 3A), confirming our previous study [15]. In contrast, while in F1 mice the LMP1/CD40-transgene caused a reduction of iTregs from 75% to 38%, in B/cDC-LMP1/CD40 mice iTregs were reduced only to 46% in comparison to 62% in B/c control mice (Fig 3A). Therefore, although in all DC-LMP1/CD40 mice iTregs were significantly reduced, B/c-animals had more iTregs left than F1- and B6-mice, which showed the strongest iTreg reduction (Fig 3A). In all mice the relative iTreg reduction was compensated by increased frequencies of nTreg, as published previously for the B6 background [15] (Fig 3A). The strain-specific differential iTreg reduction became even more evident, when the relative frequencies were calculated in correlation to background specific control littermates (Fig 3B). This analysis confirmed that iTreg frequencies were most prominently reduced in DC-LMP1/CD40-mice of the B6-background (∼20% remaining iTregs; Fig 3B), intermediately reduced in F1 (∼50% remaining iTregs; Fig 3B) and only very mildly reduced in B/c mice (∼80% remaining iTregs; Fig 3B), with a corresponding increase of nTregs, which filled the emptied Treg compartment/niche (Fig 3B).

We next determined the potential correlation between iTreg frequencies and numbers of DCs in B6, F1 and B/c mice. To this end the relative DC-frequencies of the three DC-subsets (from Fig 2B) and iTregs from the same animals (Fig 3B) were plotted (Fig 3C). The linear fit was applied to this data and the Pearson coefficient was calculated. This analysis revealed a strong positive correlation between the presence of CD103^+^ DC subsets and iTregs, while the correlation between CD103^-^CD11b^+^ DC and iTregs was negative (Fig 3C). The positive correlation between CD103+ DCs and iTregs was stronger for CD103^+^CD11b^−^ than for CD103^+^CD11b^+^ DCs, corroborating previous findings that CD103^+^CD11b^-^ DC are the most potent DC subset to induce iTregs [8].

### CD40-signalling has different background-dependent effects on DCs

**Fig 4:**
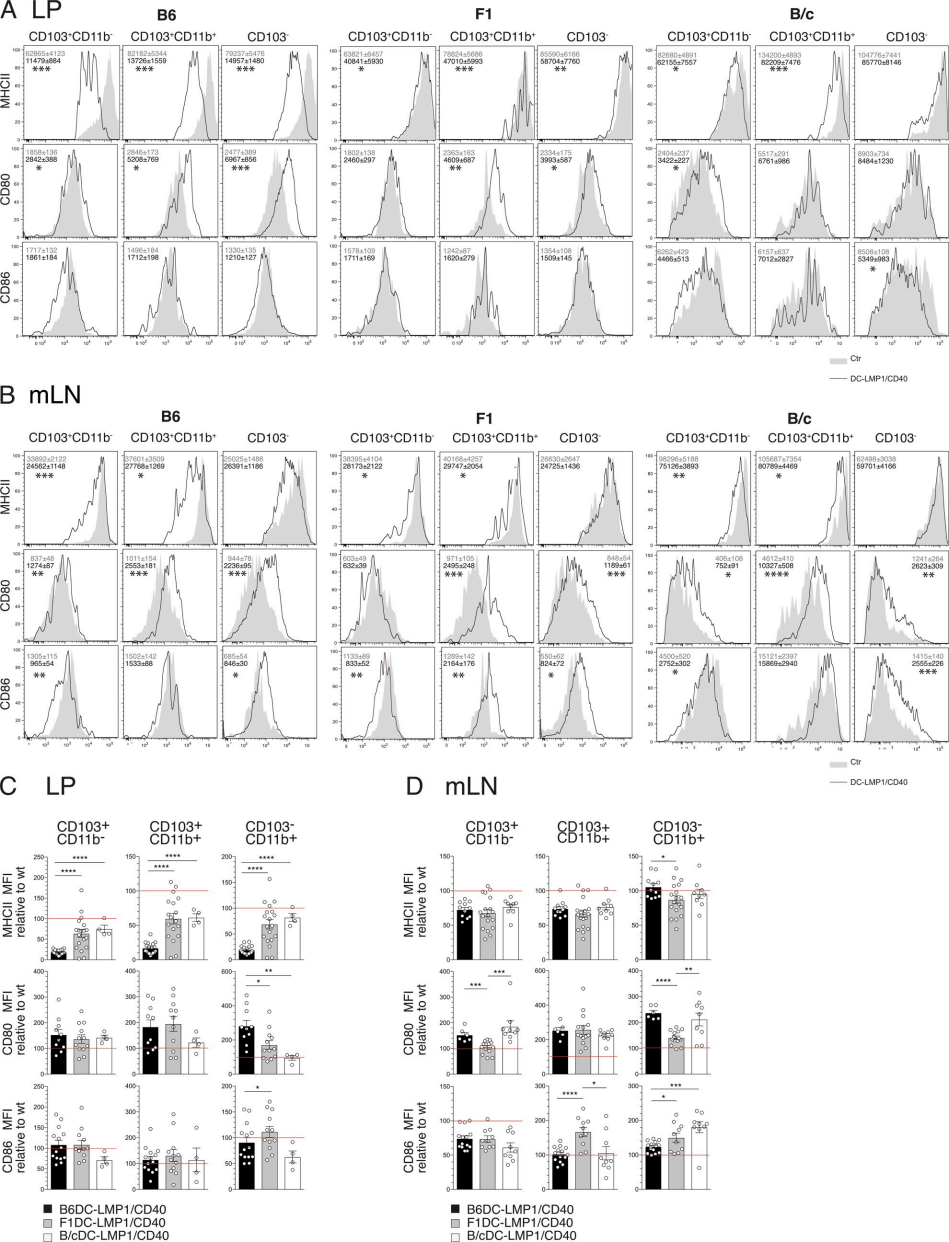
Differential maturation effects on DC-subsets in distinct backgrounds. DC subsets of the LP (A) or mLNs (B) were analyzed for the expression of different activation markers. Shown are representative histograms of wt controls (filled grey) and DC-LMP1/CD40 (black line). Numbers represent MFI ± SEM. A) Shown are pooled statistics from 5 (B6, n = 8–18), 6 (F1, n = 12–20) or 1 (B/c, n = 4–5) experiments. B) Shown are pooled statistics from 4 (B6, n = 6–14), 5 (F1, n = 10–18) or 2 (B/c, n = 9) experiments. C) The LP DC subsets frequencies from Fig 4A are shown as relative data with the corresponding background control set as 100% (indicated as red line). D) The mLN DC subsets frequencies from Fig 4B are shown as relative data with the corresponding background control set as 100% (indicated as red line). The significance was analyzed using a student’s t-test, with *: P < 0.05, **: P < 0.01, ***: P < 0.001 and ****: P < 0.0001.

It has previously been shown than TLR9-signals among other TLRs can synergize with CD40-signals [29]. As DCs from B6-mice express higher levels of TLR9 as compared to DCs from B/c-mice [30], we wondered if the signaling of the CD40-transgene in B6 mice was differentially amplified as compared to B/c-mice. To this end we compared DC-maturation markers MHCII, CD80 and CD86 in LP and mLNs of the transgenic strains on different genetic backgrounds (Fig 4). We previously observed that MHCII levels of all DC subsets from the LP of B6DC-LMP1/CD40 mice were reduced compared to DCs from non-transgenic littermates [15]. This was of relevance, as it was shown previously, that induction of iTregs depends on MHCII-expression by DCs [31]. Significantly lower MHCII levels were found on all CD103^+^ LP DCs of all three strains (Fig 4A). In mLNs these effects were more equal in DCs from all three strains, which showed reduced, but comparable MHCII expression (Fig 4B). However, comparison of expression levels in relation to wt DCs from the same genetic background showed a much stronger effect in B6 DCs as compared to those from F1 and B/c mice in LP (Fig 4C), while effects where less pronounced in mLN (Fig 4D). CD80 and CD86 were upregulated on several LP DC subsets of B6-, F1- and B/cDC-LMP1/CD40 mice (Fig 4A), yet those differences were only significant for CD103^-^ DCs, when compared relative to the corresponding wt DCs (Fig 4C). No correlation with the graded effects between B6 and B/c was found for CD80 and CD86 in LP (Fig 4C) or mLN (Fig 4D). Hence, the strongest differences which we found were distinct levels of MHCII in LP DCs.

### Differential induction of Th17, Th17/Th1 and Th1 T cells

**Fig 5:**
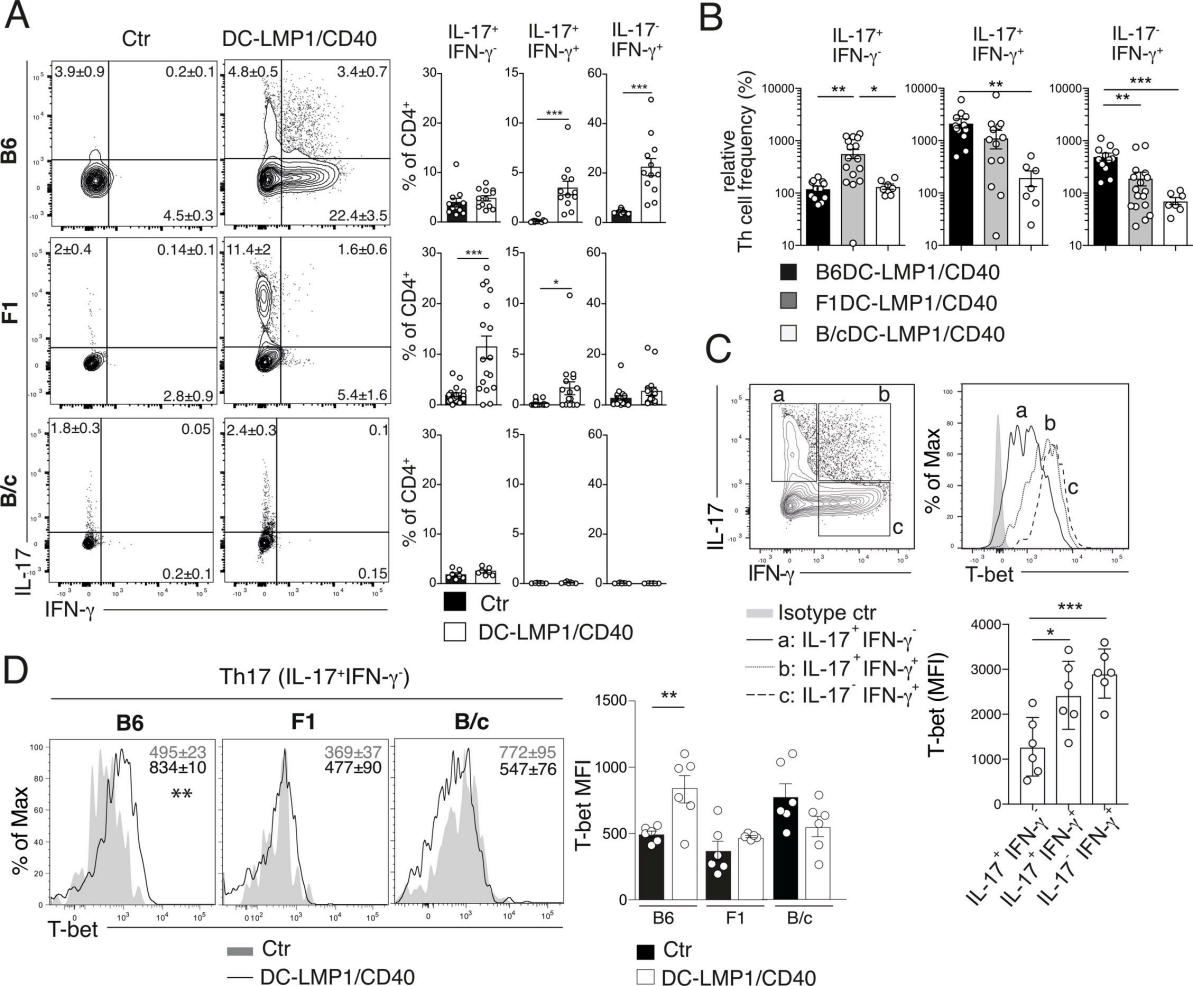
B6DC-LMP1/CD40 animals develop more IFN-γ^+^ CD4 T cells as compared to F1 or B/cDC-LMP1/CD40 animals. LP T cells were restimulated with PMA/Ionomycin for 4 h and stained intracellularly for the production of IL-17 and IFN-γ. (A) Shown are representative FACS-plots with indicated frequencies for LP (gated on single, live, CD45^+^, CD3^+^CD4^+^; see S1C Fig). Bar graphs represent the pooled statistics from 4 experiments for B6DC-LMP1/CD40 animals (n = 11–12), 6 experiments for F1DC-LMP1/CD40 animals (n = 17–18) and from 2 experiments for B/cDC-LMP1/CD40 animals (n = 7). (B) The frequencies for each Th cell subset in LP of DC-LMP1/CD40 animals on different genetic backgrounds are shown as data to relative to the corresponding background control, which was set to 100%. (C) IL-17^+^IFN-γ^-^, IL-17^+^IFN-γ^+^and IL-17^-^IFN-γ^+^ LP T cells from B6DC-LMP1/CD40 animals were analyzed for T-bet expression. The bar graph represents pooled statistics for MFI of T-bet expression from 2 pooled experiments (n = 6). (D) Shown are representative histograms of controls (grey) and DC-LMP1/CD40 (black line) for T-bet expression in IL-17^+^IFN-γ^-^ LP T cells from B6, F1 and B/cDC-LMP1/CD40 animals (grey histogram, B6, F1 or B/c controls; black line histogram, B6 (left), F1 (middle) or B/c (right) DC-LMP1/CD40 mice). The numbers on the histograms represent mean ± SEM, data from 2 experiments was pooled (n = 5–6).

Th17 cells are not a homogeneous population but can differentiate from non-pathogenic cells with important roles in host defense to pro-inflammatory cells implicated in colitis and IBD [32]. Such Th17 cell plasticity can lead to so-called “Th1-like” or “ex-Th17” cells producing IFN-γ with main roles in intestinal pathology [33–35]. Colitis in B6DC-LMP1/CD40 mice was accompanied by increased frequencies of LP-infiltrating IL-17^+^IFN-γ^+^ CD4^+^ T cells and IFN-γ^+^ Th1 cells (Fig 5A), corroborating data published previously [15]. In contrast, while F1DC-LMP1/CD40 mice also had elevated frequencies of IL-17^+^ Th17 cells in the LP, they showed only slightly elevated frequencies of IL-17^+^IFN-γ^+^ T cells and normal IFN-γ^+^ Th1 frequencies (Fig 5A). Analysis of B/cDC-LMP1/CD40 animals revealed neither differences in Th17, Th17/Th1 nor Th1 cells relative to B/c control mice (Fig 5A). Also, when the relative frequencies of the distinct Th17 and Th1 effector cell subsets were calculated in relation to the respective non-transgenic controls, B6DC-LMP1/CD40 animals had the highest IFN-γ^+^ Th1 frequencies among all three DC-LMP1/CD40 strains, while F1 transgenic animals had the highest relative frequency of IL-17^+^ Th17 T cells (Fig 5B). The effector T cell compartment in B/cDC-LMP1/CD40 animals was not different from their respective control littermates (Fig 5B). The differentiation of Th17 cells to so-called “Th1-like” or “ex-Th17” cells [36] is triggered by exposure to IL-23 [37–39] and correlates with increased expression of T-bet transcription factor [32], which controls Th1 cell differentiation [40] and transactivation of the *Ifng* gene [41]. We therefore analyzed T-bet expression in IL-17^+^IFN-γ^−^ (Th17), IL-17^+^IFN-γ^+^ (Th17/Th1) and IL-17^−^IFN-γ^+^ (Th1) T cell subsets of B6DC-LMP1/CD40 animals (Fig 5C). Here, Th17 cells expressed lowest T-bet levels, Th17^+^IFN-γ^+^ intermediate levels and Th17^−^IFN-γ^+^ cells the highest levels of T-bet (Fig 5C), reflecting their transition from Th17>Th17/Th1>Th1 cells. However, when we compared T-bet expression between IL-17^+^IFN-γ^-^ Th17 cells from B6, F1 and B/cDC-LMP1/CD40 mice, only those from the B6-background did express significantly elevated levels of T-bet (Fig 5D). This data suggests that only LP Th17 cells from B6DC-LMP1/CD40 mice have the potential for further differentiation towards the IL-17^-^IFN-γ^+^ Th1 phenotype.

### IL-1 expression in B6DCLMP1/CD40 mice is a main driver of colitis

**Fig 6:**
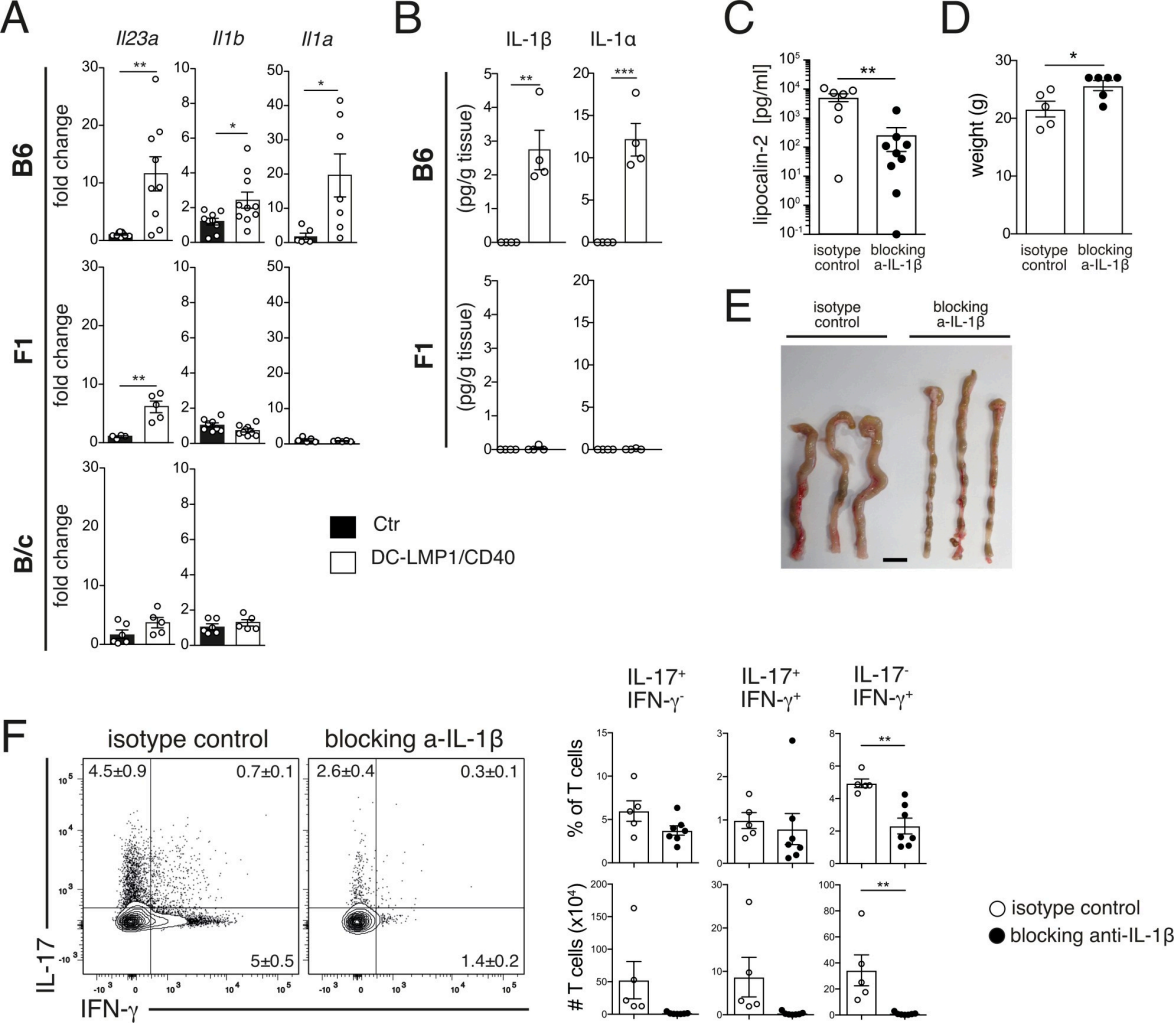
IL-1β blockade prevents colitis development in B6DC-LMP1/CD40 animals. Analysis of cytokine gene expression in LP macrophages (MPs). (A) RNA from FACS-purified CD64^+^ MPs from LP (single, live, CD45^+^MHCII^+^CD11c^+^CD64^+^ cells, for gating see S1A Fig) of B6, F1 and B/cDC-LMP1/CD40 animals were analyzed by qPCR and normalized to HPRT expression. Shown is pooled data (n = 6–10). (B) IL-1β protein was determined by ELISA in whole colon homogenates of B6 and F1DC-LMP1/CD40 or background matched Ctr animals. Shown are representative results from one of 2 independent experiments with a similar outcome (n = 4). (C, D) B6 DC-LMP1/CD40 mice were treated with blocking anti-IL-1β antibody for 7 weeks. Then, fecal Lipocalin-2 (C) and weight of animals (D) were controlled. Shown are pooled data from 2 experiments (n = 8–10). (E) Macroscopic pictures of colons from isotype control and anti-IL-1β treated B6DC-LMP1/CD40 animals after 7 weeks of therapy. Bar = 1 cm. (F) Function of LP T cells from B6DC-LMP1/CD40 animals, which were treated with anti-IL-1β mAb or isotype control, was analyzed by intracellular staining for IL-17 and IFN-γ. Shown are representative FACS-plots with indicated frequencies for LP (gating as shown in S1C Fig). Numbers in quadrants and bar graphs represent pooled data from 2 experiments (n = 5–7) with similar outcome. All bar graphs represent mean ± SEM and significance were analyzed using a student´s *t*-test, with *: *P* < 0.05, **: *P* < 0.01 and ***: *P* < 0.001.

T-bet is a key modulator of IL-23-driven intestinal colitogenic T effector functions [42]. As a source for expression of *Il23a* (encoding for IL-23p19) in B6DC-LMP1/CD40 mice we could previously identify LP CD64^+^MHCII^+^CD11c^+^ macrophages, but not CD11c^+^ DCs [15]. However, *Il23a* was also produced by LP macrophages of F1 mice (Fig 6A), which do not develop colitis (Fig 1A and 1C). Cells from mice on the B/c-background did not produce elevated levels of *Il23a* mRNA (Fig 6A). We therefore concluded that levels of IL-23 in F1DC-LMP1/CD40 mice were insufficient to induce colitis in F1 mice (Fig 1). IL-23R signals in CD4 T cells cause upregulation of IL-1R that confers pro-survival signals to T cells and contributes to their accumulation in the colon [43]. We therefore wondered if IL-1α or IL-1β, which both signal through IL-1R, were differentially expressed. Indeed, *Il1a* (encoding for IL-1α) and *Il1b* (encoding for IL-1β) were elevated in intestinal CD64^+^ macrophages from B6DC-LMP1/CD40 mice, but unaltered in mice from the F1- (Fig 6A) and B/c-background (Fig 6A, *IL1b* n.d.). Furthermore, both cytokines were elevated on the protein level in colon tissue homogenates from B6DC-LMP1/CD40-mice, but not in F1-background (Fig 6B). To find out if IL-1 was responsible for colitis development in B6DC-LMP1/CD40-mice, we next blocked IL-1β by injection of a specific blocking antibody as described previously [43]. After the treatment period, levels of lipocalin-2 in feces of anti-IL-1β treated mice were significantly reduced as compared to those receiving the isotype control antibody (Fig 6C). Anti-IL-1β treated mice had also gained significant weight (Fig 6D) without colon thickening and shortening as macroscopic signs of colitis (Fig 6E). When we analyzed T cells of the LP we found strong reduction of all three types of effector T cells, Th17, Th17/Th1 and Th1 cells in mice with IL-1β blockade (Fig 6F). This suggests that blocking of the T cell survival signal IL-1β protects B6DC-LMP1/CD40 mice from colitis and identifies IL-1β as colitis-driver, which is present in B6-, but not F1- nor B/c-background.

## Discussion

Here we demonstrate that the susceptibility to develop colitis by CD40-signalling in DCs is background-dependent. The incidence and severity of disease increased from protected B/c-mice to F1, which showed mild signs of inflammation, and culminated as fatal colitis in DC-LMP1/CD40-mice of the B6 background. This hierarchy correlated with (i) graded expression of MHCII by DC, (ii) a ranked loss of CD103^+^ DCs and (iii) differential reduction of iTreg frequencies. Furthermore, we detected differential levels of IL-1 in the different backgrounds, the blockade of which was sufficient to rescue onset of disease.

Strain-specific susceptibilities were also found in other colitis models with highly model-specific results [44]. For example, and in contrast to the data presented in this study, Rag^-/—^B/c- and -B6-mice showed no differences in colitis susceptibility upon anti-CD40 mAb-injection [45]. However, the Rag^-/-^ colitis model is T cell-independent, which is in contrast to the DC-LMP1/CD40 colitis model of the present study, where B6DC-LMP1/CD40 mice do not develop colitis when bred to the Rag^-/-^ background [15]. Similarly, exposure of mice to dextran sulfate sodium induces colitis in the absence of adaptive immunity [46]. Here, B6 mice developed stronger and more chronic effects as compared to B/c mice, the reasons for which are unclear [47]. In contrast, IL-10-mutations in the B/c-background cause more progressive disease than in the B6-background, while IL-2-mutations show stronger effects in B6 vs B/c mice [44]. The possible reasons for such variability are manifold and many differences were detected in B6 and B/c-mice [44].

DCs from B6 mice show higher expression of TLR9, the receptor for bacterial CpG, but reduced levels for TLR2, -4, -5, and -6 [30]. As TLR9 besides other TLRs has been shown to synergize with CD40-signalling in B cells [29], it is theoretically possible that a strain-specific differential TLR-expression in DCs contributes to the different grades of colitis observed in this study. It is well established that DCs project their dendrites across the epithelial layer to contact commensals and that DCs can be engaged by commensals upon their M-cell-mediated translocation [48]. Furthermore, commensals can be found inside DCs, which serve as a shuttle for their transport to mLNs [49]. Hence, there are numerous occasions when TLR-engagement might enhance CD40-signals in DCs in order to make the difference between B6 and B/c-DCs due to elevated TLR9-expression. As especially DCs of the LP barrier tissue are in close contact with commensals and TLR-ligands, these differences might contribute to colitis as observed in the DC-LMP1/CD40-model. Especially the strong MHCII downregulation in B6 vs. the other backgrounds might be an important difference. Previously, it has been shown a correlation between the numbers of DCs and Tregs. For example, Flt3 ligand was initially shown to expand Treg cells [50] and loss of Tregs causes an increased DC-division [51]. The feedback loop between DC- and Treg-numbers was also shown to depend on MHCII [52] and the removal of MHCII from DCs in vivo caused a drop in iTreg-numbers and colitis [31]. While in this latter model, colitis was not due to CD4 T cell priming as DCs lacked MHCII, in DC-LMP1/CD40-mice, MHCII is reduced but not absent. Therefore, low MHCII levels might be insufficient for iTreg induction in B6 mice but priming of CD4^+^ T cells may still be possible, as adaptive immunity is necessary for disease induction [15]. Therefore, the combination of low numbers of CD103^+^ DCs, which in addition express low levels of MHCII, might generate an environment that is insufficient for iTreg induction, causing onset of colitis.

During *Leishmania* infections, CD40-mediated host-protective functions were attributed to the generation of a Th1-bias, which could be established in B6 but not in B/c mice [53]. In general, B6 is a prototypic Th1 mouse strain with higher production of IL-12 and IFN-γ than B/c mice, which are more prone to Th2 responses [54, 55]. However, we could find elevated *Il12a* mRNA production in CD64^+^ macrophages from all three genetic backgrounds (not shown), even in F1 and B/c, which did not get sick, a finding which argues against a central role for IL-12 in our model. In addition, chronic exposure to IL-12 was reported to rather induce suppressive IL-10-production in CD4^+^ T cells [56, 57], which further decreases the possible involvement of IL-12 in colitis.

Other known strain-specific differences between B6 and B/c include different levels of polyreactive IgA abundance which impacts the generation of antigen-specific IgA and microbiota diversity. B/c mice showed higher IgA abundance, which was directly responsible for a higher microbiota diversity [58]. As colitis in DC-LMP1/CD40-mice is dependent on microbiota, also these differences might be relevant for disease onset. Although all strains were cohoused in the same room of the animal facility, we did not determine microbiota diversity in the present study.

Strain-specific plasticity in T cell differentiation could be another causative key factor in the present study. Th17 cells are rather protective as they also produce anti-inflammatory IL-10 [59]. Further exposure to IL-23 results in a pathogenic expression signature, which in addition to T-bet, the master regulator of Th1 cells, includes GM-CSF, IL-23R and IL-7R [37, 38]. IL-17 expression of Th17 cells is transient in some inflammatory settings, because after IL-23 conversion cells can stop expressing IL-17, acquire the ability to express IFN-γ to become “ex-Th17” or “Th1-like” cells [36]. Also, exposure to a combination of IL-1, IL-6 and IL-23 can induce pathogenic Th17 cells [38, 60]. IL-23R-signalling is necessary for induction of intestinal T cell pathogenicity [61] and IL-1R is upregulated in Th17 cells [62]. Downregulation of IL-1R and -23R interferes with pathogenicity of Th17 cells [63], underlining the role of both cytokines in pathogenic differentiation of Th17 cells [32].

IL-10 signaling does suppress IL-1β secretion [64] and IL-1β drives T cell effector responses [43]. This might be of relevance for the colitis model presented here, as the levels of IL-10 were significantly reduced in the colon of B6 DC-LMP1/CD40 mice, but not those of other backgrounds. Macrophages require IL-10 signaling for maintenance of gut homeostasis [65, 66]. In absence of IL-10 signals, intestinal macrophages produce elevated levels of IL-1β [67]. We found differential expression of IL-1 in B6 vs. F1 mice and antibody blockade of IL-1 rescued mice on the B6-background from onset of colitis by reducing CD4 T cell numbers and avoiding generation of Th17/Th1 and Th1 cells. Therefore, differential IL-1 production might be central to colitis progression in this model. In B6 we found elevated levels of *Il1b* and *Il23a* mRNA, which would support such a preferential ex-Th17 differentiation. In contrast, in F1 mice only *Il23a*, but neither *Il1a nor Il1b* mRNA was changed and cells with an “ex-Th17”-phenotype were not elevated. As IL-23R signals are central to upregulation of IL-1R by Th17 cells and IL-1R confers pro-survival signals for accumulation of T cells in the colon [43], the IL-23/IL-1 axis plays most likely an important role for the differences seen in the DC-LMP1/CD40-model for colitis. However, we have shown previously that *Il23a* and *Il1b* mRNA is not induced directly by the LMP1/CD40-transgene, but rather a secondary effect of inflammation, as in B6DC-LMP1/CD40-mice on the Rag-ko background, these cytokines are not present and mice do not develop colitis [15]. Therefore, most likely bystander effects from the innate immune system, which might contribute to the overall sensing of commensals, or microbe handling can eventually determine the outcome of CD40-signalling in the DC-LMP1/CD40-model in B6-, F1- or B/c-backgrounds. However, further studies will be necessary to identify determinants responsible the observed differences.

## Supporting information

S1 Fig Gating strategies used for FACS analyses. (A) Gating strategy for the identification of macrophages (MPs; single cells, live, CD45^+^, MHCII^+^CD11c^+^, CD64^+^ cells) and DCs (single cells, live, CD45^+^, MHCII^+^CD11c^+^, CD64^-^ cells) in LP (upper panel) and DC in mLN (lower panel) of experimental mice. (B) Gating strategy for the identification of Treg subsets in LP of experimental mice (single cells, live, CD45^+^CD3^+^CD4^+^CD25^+^FoxP3^+^Helios^+^ nTregs or Helios^-^ iTregs). (C) Gating for the identification of CD4^+^ T helper cell subsets in LP of experimental mice (single cells, live, CD45^+^, CD3^+^CD4^+^, IL-17^+^IFN-γ^-^ / IL-17^+^IFN-γ^+^ / IL-17^-^IFN-γ^+^ cells).
(TIFF)

S2 Fig Graded loss of CD103^+^ DCs from the mLN of DC-LMP1/CD40 animals. DC subsets in the mLNs were analysed in DC-LMP1/CD40 animals on different genetic backgrounds. mLN cells were pre-gated on single cells, live, CD45^+^, MHCII^+^CD11c^+^, CD64^-^ cells from control (Ctr) or DC-LMP1/CD40 mice on B6-, F1- or B/c-background. Representative FACS-plots are shown. Numbers and bar graphs indicate the frequencies of DC subsets within the gates. Shown is pooled data from 4 (B6, n = 12–14), 5 (F1; n = 18) or 2 experiments (B/c, n = 6–7) with similar outcome. All bar graphs represent mean ± SEM where significance was analyzed using a student´s *t*-test, with *: P < 0.05, **: P < 0.01 and ***: P < 0.001.
(TIFF)

## References

[1] Sakaguchi S, Yamaguchi T, Nomura T, Ono M. Regulatory T cells and immune tolerance. Cell. 2008;133(5):775–87. 10.1016/j.cell.2008.05.009 18510923

[2] Chen W, Jin W, Hardegen N, Lei KJ, Li L, Marinos N, et al Conversion of peripheral CD4+CD25- naive T cells to CD4+CD25+ regulatory T cells by TGF-beta induction of transcription factor Foxp3. J Exp Med. 2003;198(12):1875–86. 10.1084/jem.20030152 14676299 PMC2194145

[3] Ai TL, Solomon BD, Hsieh CS. T-cell selection and intestinal homeostasis. Immunol Rev. 2014;259(1):60–74. 10.1111/imr.12171 24712459 PMC4028094

[4] Josefowicz SZ, Lu LF, Rudensky AY. Regulatory T cells: mechanisms of differentiation and function. Annu Rev Immunol. 2012;30:531–64. 10.1146/annurev.immunol.25.022106.141623 22224781 PMC6066374

[5] Ohnmacht C, Park JH, Cording S, Wing JB, Atarashi K, Obata Y, et al The microbiota regulates type 2 immunity through RORgammat(+) T cells. Science. 2015;349(6251):989–93. 10.1126/science.aac4263 26160380

[6] Sefik E, Geva-Zatorsky N, Oh S, Konnikova L, Zemmour D, McGuire AM, et al Individual intestinal symbionts induce a distinct population of RORgamma(+) regulatory T cells. Science. 2015;349(6251):993–7. 10.1126/science.aaa9420 26272906 PMC4700932

[7] Coombes JL, Siddiqui KR, Arancibia-Carcamo CV, Hall J, Sun CM, Belkaid Y, et al A functionally specialized population of mucosal CD103+ DCs induces Foxp3+ regulatory T cells via a TGF-{beta} and retinoic acid dependent mechanism. J Exp Med. 2007;204(8):1757–64. 10.1084/jem.20070590 17620361 PMC2118683

[8] Esterhazy D, Loschko J, London M, Jove V, Oliveira TY, Mucida D. Classical dendritic cells are required for dietary antigen-mediated induction of peripheral Treg cells and tolerance. Nat Immunol. 2016;17(5):545–55. 10.1038/ni.3408 27019226 PMC4837106

[9] Sun CM, Hall JA, Blank RB, Bouladoux N, Oukka M, Mora JR, et al Small intestine lamina propria dendritic cells promote de novo generation of Foxp3 T reg cells via retinoic acid. J Exp Med. 2007;204(8):1775–85. 10.1084/jem.20070602 17620362 PMC2118682

[10] Cerovic V, Houston SA, Scott CL, Aumeunier A, Yrlid U, Mowat AM, et al Intestinal CD103(-) dendritic cells migrate in lymph and prime effector T cells. Mucosal Immunol. 2013;6(1):104–13. 10.1038/mi.2012.53 22718260

[11] Lewis KL, Caton ML, Bogunovic M, Greter M, Grajkowska LT, Ng D, et al Notch2 receptor signaling controls functional differentiation of dendritic cells in the spleen and intestine. Immunity. 2011;35(5):780–91. 10.1016/j.immuni.2011.08.013 22018469 PMC3225703

[12] Persson EK, Uronen-Hansson H, Semmrich M, Rivollier A, Hagerbrand K, Marsal J, et al IRF4 transcription-factor-dependent CD103(+)CD11b(+) dendritic cells drive mucosal T helper 17 cell differentiation. Immunity. 2013;38(5):958–69. 10.1016/j.immuni.2013.03.009 23664832

[13] Uematsu S, Fujimoto K, Jang MH, Yang BG, Jung YJ, Nishiyama M, et al Regulation of humoral and cellular gut immunity by lamina propria dendritic cells expressing Toll-like receptor 5. Nat Immunol. 2008;9(7):769–76. 10.1038/ni.1622 18516037

[14] Cerovic V, Bain CC, Mowat AM, Milling SW. Intestinal macrophages and dendritic cells: what's the difference? Trends Immunol. 2014;35(6):270–7. 10.1016/j.it.2014.04.003 24794393

[15] Barthels C, Ogrinc A, Steyer V, Meier S, Simon F, Wimmer M, et al CD40-signalling abrogates indþuction of RORgt+ Treg cells by intestinal CD103 DCs and causes fatal colitis. Nat Commun. 2017;8:14715 10.1038/ncomms14715 28276457 PMC5347138

[16] Danese S, Katz JA, Saibeni S, Papa A, Gasbarrini A, Vecchi M, et al Activated platelets are the source of elevated levels of soluble CD40 ligand in the circulation of inflammatory bowel disease patients. Gut. 2003;52(10):1435–41. 12970136 10.1136/gut.52.10.1435 PMC1773814

[17] Liu Z, Colpaert S, D'Haens GR, Kasran A, de Boer M, Rutgeerts P, et al Hyperexpression of CD40 ligand (CD154) in inflammatory bowel disease and its contribution to pathogenic cytokine production. J Immunol. 1999;163(7):4049–57. 10491009

[18] Ludwiczek O, Kaser A, Tilg H. Plasma levels of soluble CD40 ligand are elevated in inflammatory bowel diseases. Int J Colorectal Dis. 2003;18(2):142–7. 10.1007/s00384-002-0425-4 12548417

[19] Gonsky R, Deem RL, C.J. L, Haritunians T, Yang S, Targan SR. IFNG rs1861494 polymorphism is associated with IBD disease severity and functional changes in both IFNG methylation and protein secretion. Inflamm Bowel Dis. 2014;20(10):1794–801. 10.1097/MIB.0000000000000172 25171510 PMC4327845

[20] Danese S, Sans M, Fiocchi C. The CD40/CD40L costimulatory pathway in inflammatory bowel disease. Gut. 2004;53:1035–43. 10.1136/gut.2003.026278 15194658 PMC1774101

[21] Hart AL, Al-Hassi HO, Rigby RJ, Bell SJ, Emmanuel AV, Knight SC, et al Characteristics of intestinal dendritic cells in inflammatory bowel diseases. Gastroenterology. 2005;129(1):50–65. 16012934 10.1053/j.gastro.2005.05.013

[22] Liu Z, Geboes K, Colpaert S, Overbergh L, Mathieu C, Heremans H, et al Prevention of Experimental Colitis in SCID Mice Reconstituted with CD45RB high CD4+ T Cells by Blocking the CD40-CD154 Interactions. J Immunol. 2000;164:6005–14. 10820284 10.4049/jimmunol.164.11.6005

[23] Stuber E, Strober W, Neurath M. Blocking the CD40L-CD40 interaction in vivo specifically prevents the priming of T helper 1 cells through the inhibition of interleukin 12 secretion. J Exp Med. 1996;183(2):693–8. 8627184 10.1084/jem.183.2.693 PMC2192468

[24] Kasran A, Boon L, Wortel CH, Hogezand RA, Schreiber S, Goldin E, et al Safety and tolerability of antagonist anti-human CD40 Mab ch5D12 in patients with moderate to severe Crohn's disease. Aliment Pharmacol Ther. 2005;22(2):111–22. 10.1111/j.1365-2036.2005.02526.x 16011669

[25] Caton ML, Smith-Raska MR, Reizis B. Notch-RBP-J signaling controls the homeostasis of CD8- dendritic cells in the spleen. J Exp Med. 2007;204(7):1653–64. 10.1084/jem.20062648 17591855 PMC2118632

[26] Homig-Holzel C, Hojer C, Rastelli J, Casola S, Strobl LJ, Muller W, et al Constitutive CD40 signaling in B cells selectively activates the noncanonical NF-kappaB pathway and promotes lymphomagenesis. J Exp Med. 2008;205(6):1317–29. 10.1084/jem.20080238 18490492 PMC2413030

[27] Hurdayal R, Nieuwenhuizen NE, Revaz-Breton M, Smith L, Hoving JC, Parihar SP, et al Deletion of IL-4 receptor alpha on dendritic cells renders BALB/c mice hypersusceptible to Leishmania major infection. PLoS Pathog. 2013;9(10):e1003699 10.1371/journal.ppat.1003699 24204259 PMC3812013

[28] Chassaing B, Srinivasan G, Delgado MA, Young AN, Gewirtz AT, Vijay-Kumar M. Fecal lipocalin 2, a sensitive and broadly dynamic non-invasive biomarker for intestinal inflammation. PLoS One. 2012;7(9):e44328 10.1371/journal.pone.0044328 22957064 PMC3434182

[29] Boeglin E, Smulski CR, Brun S, Milosevic S, Schneider P, Fournel S. Toll-like receptor agonists synergize with CD40L to induce either proliferation or plasma cell differentiation of mouse B cells. PLoS One. 2011;6(10):e25542 10.1371/journal.pone.0025542 21991317 PMC3184999

[30] Liu T, Matsuguchi T, Tsuboi N, Yajima T, Yoshikai Y. Differences in expression of toll-like receptors and their reactivities in dendritic cells in BALB/c and C57BL/6 mice. Infect Immun. 2002;70(12):6638–45. 10.1128/IAI.70.12.6638-6645.2002 12438336 PMC133006

[31] Loschko J, Schreiber HA, Rieke GJ, Esterhazy D, Meredith MM, Pedicord VA, et al Absence of MHC class II on cDCs results in microbial-dependent intestinal inflammation. J Exp Med. 2016;213(4):517–34. 10.1084/jem.20160062 27001748 PMC4821651

[32] Stockinger B, Omenetti S. The dichotomous nature of T helper 17 cells. Nat Rev Immunol. 2017;17(9):535–44. 10.1038/nri.2017.50 28555673

[33] Ahlfors H, Morrison PJ, Duarte JH, Li Y, Biro J, Tolaini M, et al IL-22 fate reporter reveals origin and control of IL-22 production in homeostasis and infection. J Immunol. 2014;193(9):4602–13. 10.4049/jimmunol.1401244 25261485 PMC4201943

[34] Harbour SN, Maynard CL, Zindl CL, Schoeb TR, Weaver CT. Th17 cells give rise to Th1 cells that are required for the pathogenesis of colitis. Proc Natl Acad Sci U S A. 2015;112(22):7061–6. 10.1073/pnas.1415675112 26038559 PMC4460486

[35] Morrison PJ, Bending D, Fouser LA, Wright JF, Stockinger B, Cooke A, et al Th17-cell plasticity in Helicobacter hepaticus–induced intestinal inflammation. Mucosal Immunology. Mucosal Immunol. 2013;6(6):1143–56. 10.1038/mi.2013.11 23462910

[36] Hirota K, Duarte JH, Veldhoen M, Hornsby E, Li Y, Cua DJ, et al Fate mapping of IL-17-producing T cells in inflammatory responses. Nat Immunol. 2011;12(3):255–63. 10.1038/ni.1993 21278737 PMC3040235

[37] Langrish CL, Chen Y, Blumenschein WM, Mattson J, Basham B, Sedgwick JD, et al IL-23 drives a pathogenic T cell population that induces autoimmune inflammation. J Exp Med. 2005;201(2):233–40. 10.1084/jem.20041257 15657292 PMC2212798

[38] Lee Y, Awasthi A, Yosef N, Quintana FJ, Xiao S, Peters A, et al Induction and molecular signature of pathogenic TH17 cells. Nat Immunol. 2012;13(10):991–9. 10.1038/ni.2416 22961052 PMC3459594

[39] Peters A, Lee Y, Kuchroo VK. The many faces of Th17 cells. Curr Opin Immunol. 2011;23(6):702–6. 10.1016/j.coi.2011.08.007 21899997 PMC3232281

[40] Szabo SJ, Kim ST, Costa GL, Zhang X, Fathman CG, Glimcher LH. A novel transcription factor, T-bet, directs Th1 lineage commitment. Cell. 2000;100(6):655–69. 10761931 10.1016/s0092-8674(00)80702-3

[41] Zhu J, Jankovic D, Oler AJ, Wei G, Sharma S, Hu G, et al The transcription factor T-bet is induced by multiple pathways and prevents an endogenous Th2 cell program during Th1 cell responses. Immunity. 2012;37(4):660–73. 10.1016/j.immuni.2012.09.007 23041064 PMC3717271

[42] Krausgruber T, Schiering C, Adelmann K, Harrison OJ, Chomka A, Pearson C, et al T-bet is a key modulator of IL-23-driven pathogenic CD4(+) T cell responses in the intestine. Nat Commun. 2016;7:11627 10.1038/ncomms11627 27193261 PMC4874038

[43] Coccia M, Harrison OJ, Schiering C, Asquith MJ, Becher B, Powrie F, et al IL-1β mediates chronic intestinal inflammation by promoting the accumulation of IL-17A secreting innate lymphoid cells and CD4(+) Th17 cells. J Exp Med. 2012;209(9):1595–15609. 10.1084/jem.20111453 22891275 PMC3428945

[44] Sellers RS, Clifford CB, Treuting PM, Brayton C. Immunological Variation Between Inbred Laboratory Mouse Strains: Points to Consider in Phenotyping Genetically Immunomodified Mice. Veterinary Pathology. 2012;49(1):32–43. 10.1177/0300985811429314 22135019

[45] van Driel B, Liao G, Romero X, O'Keeffe MS, Wang G, Faubion WA, et al Signaling lymphocyte activation molecule regulates development of colitis in mice. Gastroenterology. 2012;143(6):1544–54. 10.1053/j.gastro.2012.08.042 22960654 PMC3578298

[46] Dieleman LA, Ridwan BU, Tennyson GS, Beagley KW, Bucy RP, Elson CO. Dextran sulfate sodium-induced colitis occurs in severe combined immunodeficient mice. Gastroenterology. 1994;107(6):1643–52. 7958674 10.1016/0016-5085(94)90803-6

[47] Melgar S, Karlsson A, Michaelsson E. Acute colitis induced by dextran sulfate sodium progresses to chronicity in C57BL/6 but not in BALB/c mice: correlation between symptoms and inflammation. Am J Physiol Gastrointest Liver Physiol. 2005;288(6):G1328–38. 10.1152/ajpgi.00467.2004 15637179

[48] Coombes JL, Powrie F. Dendritic cells in intestinal immune regulation. Nat Rev Immunol. 2008;8(6):435–46. 10.1038/nri2335 18500229 PMC2674208

[49] Macpherson AJ, Uhr T. Induction of protective IgA by intestinal dendritic cells carrying commensal bacteria. Science. 2004;303(5664):1662–5. 10.1126/science.1091334 15016999

[50] Swee LK, Bosco N, Malissen B, Ceredig R, Rolink A. Expansion of peripheral naturally occurring T regulatory cells by Fms-like tyrosine kinase 3 ligand treatment. Blood. 2009;113(25):6277–87. 10.1182/blood-2008-06-161026 19211508

[51] Liu K, Victora GD, Schwickert TA, Guermonprez P, Meredith MM, Yao K, et al In vivo analysis of dendritic cell development and homeostasis. Science. 2009;324(5925):392–7. 10.1126/science.1170540 19286519 PMC2803315

[52] Darrasse-Jeze G, Deroubaix S, Mouquet H, Victora GD, Eisenreich T, Yao KH, et al Feedback control of regulatory T cell homeostasis by dendritic cells in vivo. J Exp Med. 2009;206(9):1853–62. 10.1084/jem.20090746 19667061 PMC2737156

[53] Awasthi A, Mathur R, Khan A, Joshi BN, Jain N, Sawant S, et al CD40 signaling is impaired in L. major-infected macrophages and is rescued by a p38MAPK activator establishing a host-protective memory T cell response. J Exp Med. 2003;197(8):1037–43. 10.1084/jem.20022033 12695487 PMC2193877

[54] Mills CD, Kincaid K, Alt JM, Heilman MJ, Hill AM. M-1/M-2 macrophages and the Th1/Th2 paradigm. J Immunol. 2000;164(12):6166–73. 10843666 10.4049/jimmunol.164.12.6166

[55] Watanabe H, Numata K, Ito T, Takagi K, Matsukawa A. Innate immune response in Th1- and Th2-dominant mouse strains. Shock. 2004;22(5):460–6. 15489639 10.1097/01.shk.0000142249.08135.e9

[56] Gerosa F, Paganin C, Peritt D, Paiola F, Scupoli MT, Aste-Amezaga M, et al Interleukin-12 primes human CD4 and CD8 T cell clones for high production of both interferon-gamma and interleukin-10. J Exp Med. 1996;183(6):2559–69. 8676077 10.1084/jem.183.6.2559 PMC2192598

[57] Trinchieri G. Interleukin-10 production by effector T cells: Th1 cells show self control. J Exp Med. 2007;204(2):239–43. 10.1084/jem.20070104 17296790 PMC2118719

[58] Fransen F, Zagato E, Mazzini E, Fosso B, Manzari C, El Aidy S, et al BALB/c and C57BL/6 Mice Differ in Polyreactive IgA Abundance, which Impacts the Generation of Antigen-Specific IgA and Microbiota Diversity. Immunity. 2015;43(3):527–40. 10.1016/j.immuni.2015.08.011 26362264

[59] McGeachy MJ, Bak-Jensen KS, Chen Y, Tato CM, Blumenschein W, McClanahan T, et al TGF-beta and IL-6 drive the production of IL-17 and IL-10 by T cells and restrain T(H)-17 cell-mediated pathology. Nat Immunol. 2007;8(12):1390–7. 10.1038/ni1539 17994024

[60] Ghoreschi K, Laurence A, Yang XP, Tato CM, McGeachy MJ, Konkel JE, et al Generation of pathogenic T(H)17 cells in the absence of TGF-beta signalling. Nature. 2010;467(7318):967–71. 10.1038/nature09447 20962846 PMC3108066

[61] Ahern PP, Schiering C, Buonocore S, McGeachy MJ, Cua DJ, Maloy KJ, et al Interleukin-23 drives intestinal inflammation through direct activity on T cells. Immunity. 2010;33(2):279–88. 10.1016/j.immuni.2010.08.010 20732640 PMC3078329

[62] Chung Y, Chang SH, Martinez GJ, Yang XO, Nurieva R, Kang HS, et al Critical regulation of early Th17 cell differentiation by interleukin-1 signaling. Immunity. 2009;30(4):576–87. 10.1016/j.immuni.2009.02.007 19362022 PMC2705871

[63] Kishi Y, Kondo T, Xiao S, Yosef N, Gaublomme J, Wu C, et al Protein C receptor (PROCR) is a negative regulator of Th17 pathogenicity. J Exp Med. 2016;213(11):2489–501. 10.1084/jem.20151118 27670590 PMC5068226

[64] Guarda G, Braun M, Staehli F, Tardivel A, Mattmann C, Forster I, et al Type I interferon inhibits interleukin-1 production and inflammasome activation. Immunity. 2011;34(2):213–23. 10.1016/j.immuni.2011.02.006 21349431

[65] Zigmond E, Bernshtein B, Friedlander G, Walker CR, Yona S, Kim KW, et al Macrophage-restricted interleukin-10 receptor deficiency, but not IL-10 deficiency, causes severe spontaneous colitis. Immunity. 2014;40(5):720–33. 10.1016/j.immuni.2014.03.012 24792913

[66] Shouval DS, Biswas A, Goettel JA, McCann K, Conaway E, Redhu NS, et al Interleukin-10 receptor signaling in innate immune cells regulates mucosal immune tolerance and anti-inflammatory macrophage function. Immunity. 2014;40(5):706–19. 10.1016/j.immuni.2014.03.011 24792912 PMC4513358

[67] Shouval DS, Biswas A, Kang YH, Griffith AE, Konnikova L, Mascanfroni ID, et al Interleukin 1beta Mediates Intestinal Inflammation in Mice and Patients With Interleukin 10 Receptor Deficiency. Gastroenterology. 2016;151(6):1100–4. 10.1053/j.gastro.2016.08.055 27693323 PMC5124405

