# Supplementary figures and images for: Strain specific maturation of Dendritic cells and production of IL-1β controls CD40-driven colitis
Source: PLoS One. 2019 Jan 17;14(1):e0210998. doi: 10.1371/journal.pone.0210998 (PMC6336277; doi:10.1371/journal.pone.0210998)

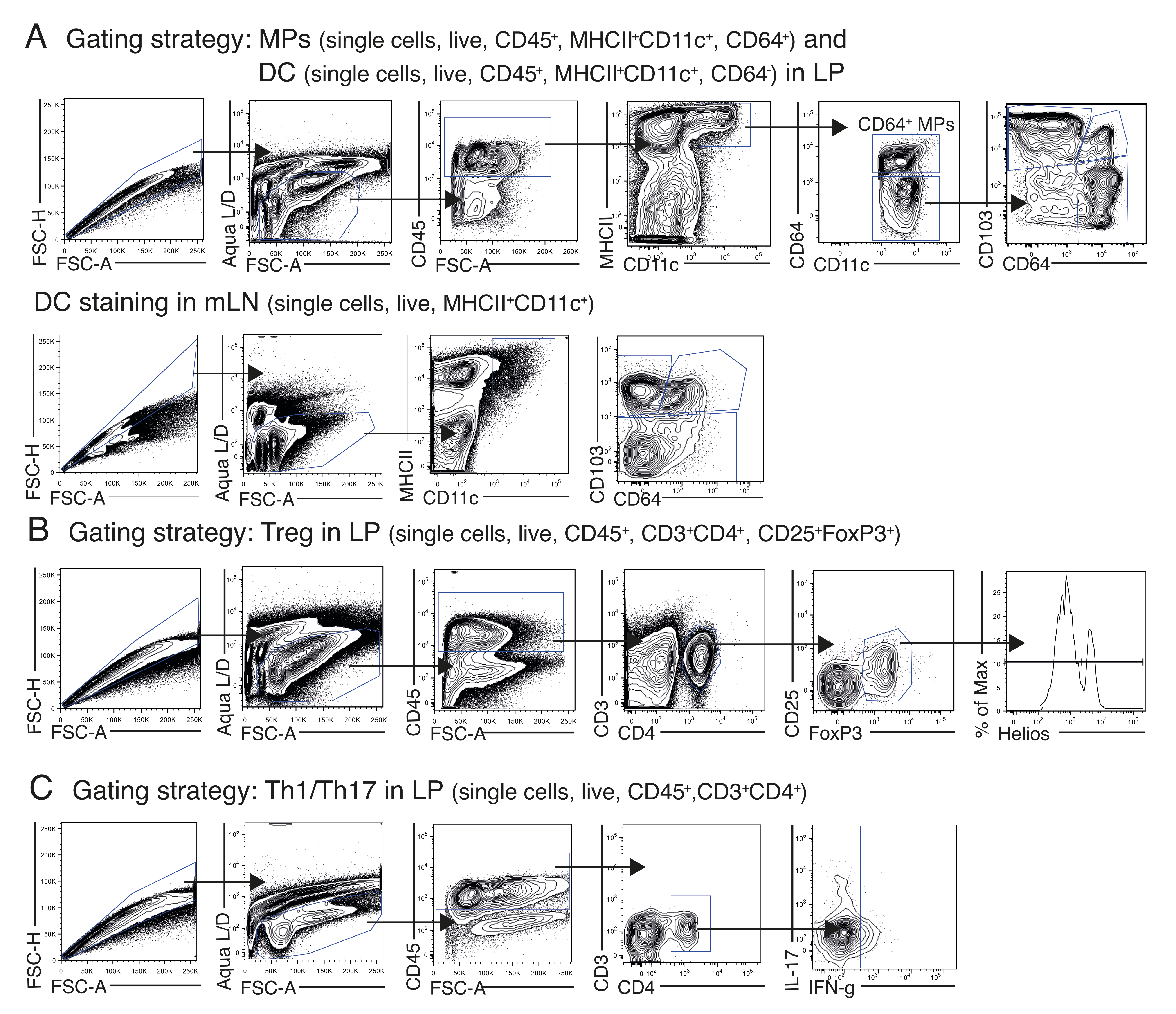

Supplement: S1 Fig — (A) Gating strategy for the identification of macrophages (MPs; single cells, live, CD45+, MHCII+CD11c+, CD64+ cells) and DCs (single cells, live, CD45+, MHCII+CD11c+, CD64- cells) in LP (upper panel) and DC in mLN (lower panel) of experimental mice. (B) Gating strategy for the identification of Treg subsets in LP of experimental mice (single cells, live, CD45+CD3+CD4+CD25+FoxP3+Helios+ nTregs or Helios- iTregs). (C) Gating for the identification of CD4+ T helper cell subsets in LP of experimental mice (single cells, live, CD45+, CD3+CD4+, IL-17+IFN-γ- / IL-17+IFN-γ+ / IL-17-IFN-γ+ cells). (TIFF) [file pone.0210998.s001.tiff]

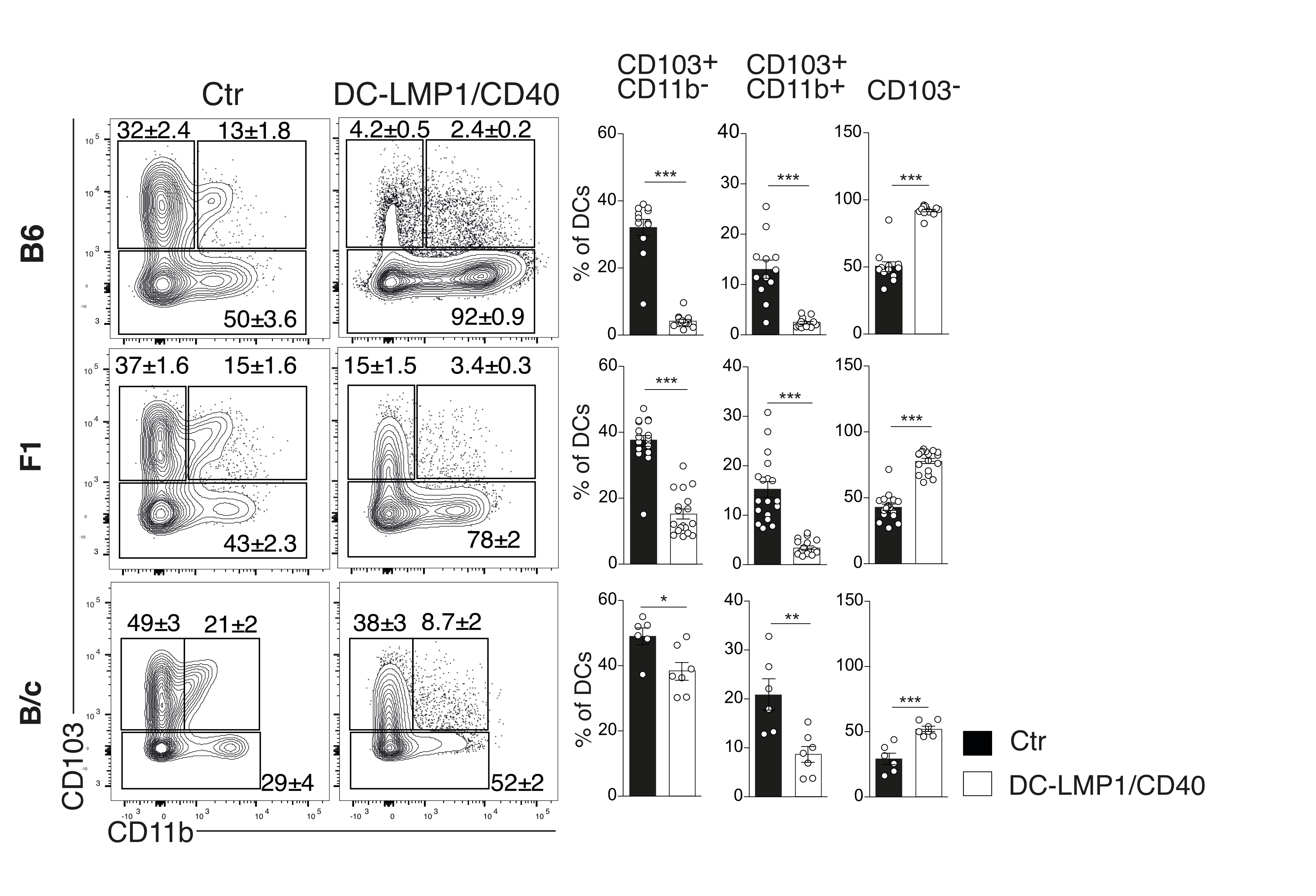

Supplement: S2 Fig — DC subsets in the mLNs were analysed in DC-LMP1/CD40 animals on different genetic backgrounds. mLN cells were pre-gated on single cells, live, CD45+, MHCII+CD11c+, CD64- cells from control (Ctr) or DC-LMP1/CD40 mice on B6-, F1- or B/c-background. Representative FACS-plots are shown. Numbers and bar graphs indicate the frequencies of DC subsets within the gates. Shown is pooled data from 4 (B6, n = 12–14), 5 (F1; n = 18) or 2 experiments (B/c, n = 6–7) with similar outcome. All bar graphs represent mean ± SEM where significance was analyzed using a student´s t-test, with *: P < 0.05, **: P < 0.01 and ***: P < 0.001. (TIFF) [file pone.0210998.s002.tiff]
